# Supplementary material for: Plasma neurofilament light protein correlates with diffusion tensor imaging metrics in frontotemporal dementia
Source: PLoS One. 2020 Oct 27;15(10):e0236384. doi: 10.1371/journal.pone.0236384 (PMC7591030; doi:10.1371/journal.pone.0236384)
Supplement: S1 Appendix — (DOCX) [file pone.0236384.s001.docx]

**S1 appendix: Tracts of interest analysis: Comparison of FA values between bvFTD and healthy controls**

The differences in DTI metrics between bvFTD and healthy controls were tested with ANCOVA including age as a nuisance covariate. The analysis showed a significant reduction of FA values in the bvFTD group relative to healthy controls in all the tracts of interests except in the the inferior cingulum bundle [lUF: F(1,39)=30.2, FDR-p < 0.001; rUF: F(1,39)=30.6, FDR-p< 0.001; lFOF: F(1,39)=22.0, FDR-p<0.001; rFOF: F(1,39)=25.6, FDR-p<0.001; lCST: F(1,39)=11.2, FDR-p<0.01; rCST: F(1,39)=7.0, FDR-p<0.05; lATR: F(1,39)=12.8, FDR-p<0.01; rATR: F(1,39)=17.4, FDR-p<0.001; ldCin: F(1,39)=13.6, FDR-p<0.01; rdCin: F(1,39)=14.8, p<0.001; lSLF: F(1,39)=14.4, FDR-p<0.001; rSLF: F(1,39)=18.1, FDR-p<0.001; liCin: F(1,39)=1.7, p>0.2; riCin: F(1,39)=2.0, p>0.1].
