# Supplementary material for: Plasma neurofilament light protein correlates with diffusion tensor imaging metrics in frontotemporal dementia
Source: PLoS One. 2020 Oct 27;15(10):e0236384. doi: 10.1371/journal.pone.0236384 (PMC7591030; doi:10.1371/journal.pone.0236384)
Supplement: S2 Appendix — (DOCX) [file pone.0236384.s002.docx]

**S2 appendix: Tracts of interest analysis: Association between FA and plasma NfL levels in the tracts that exhibited a reduction in FA in the bvFTD cohort**

Considering the lack of significant results in the group comparison the iCin was not further tested.

- lFOF – group: β=-0.03, p<0.05, FDR-p<0.05, 95% CI: -0.059 – -0.005; age: β=-0.002, p=0.08, 95% CI: -0.004 – 0.
- rFOF – group: β=-0.03, p<0.05, FDR-p<0.05, 95% CI: -0.062 – -0.006; age: β=-0.001, p>0.4, 95% CI: -0.003 – 0.1.
- lSLF – group: β=-0.03, p<0.05, FDR-p<0.05, 95% CI: -0.049 – -0.006; age: β=-0.002, p<0.05, 95% CI: -0.003 – 0.
- rSLF – group: β=-0.03, p<0.05, FDR-p<0.05, 95% CI: -0.057 – -0.005; age: β=-0.001, p>0.1, 95% CI: -0.003 – 0.001].
- lUF – group: β=-0.03 , p<0.05, FDR-p < 0.05, 95% CI: -0.065 – -0.004; age: β=-0.001, p>0.2, 95% CI: -0.003 – 0.001.
- rUF – group: β=-0.04 , p=0.06, 95% CI: -0.071 – 0.001; age: β=-0.001, p>0.5, 95% CI: -0.003 – 0.002.
- lATR – group: β=-0.02 , p=0.06, 95% CI: -0.048 – 0.002; age: β=-0.003, p<0.01, 95% CI: -0.004 – -0.001.
- rATR – group: β=-0.04, p<0.01, FDR-p<0.05, 95% CI: -0.062 – -0.011; age: β=-0.002, p<0.05, 95% CI: -0.004 – -4.67e^-06.^
- ldCin – group: β=-0.03, p=0.05, 95% CI: -0.057 – -6.51e-05; age: β=-0.002, p<0.05, 95% CI: -0.004 – 0.
- rdCin – group: β=-0.04, p<0.05, FDR-p<0.05, 95% CI: -0.074 – 0.008; age: β=-0.001, p>0.2, 95% CI: -0.004 – 0.001.
- lCST – group: β=-0.01, p>0.1, 95% CI: -0.029 – 0.005; age: β=-0.001, p<0.05, 95% CI: -0.003 – -7.44e^-05.^
- rCST – group: β=-0.01, p>0.1, 95% CI: -0.033 – 0.004; age: β=-0.001, p<0.04, 95% CI: -0.003 – -4.58e^-05^.
